# An Asymmetrical Glycerol Diether Bolalipid with Protonable Phosphodimethylethanolamine Headgroup: The Impact of pH on Aggregation Behavior and Miscibility with DPPC

**DOI:** 10.3390/polym9110573

**Published:** 2017-11-03

**Authors:** Thomas Markowski, Sindy Müller, Bodo Dobner, Annette Meister, Alfred Blume, Simon Drescher

**Affiliations:** 1 Institute of Pharmacy—Biochemical Pharmacy, Martin Luther University (MLU) Halle-Wittenberg, Wolfgang-Langenbeck-Strasse 4, 06120 Halle (Saale), Germany; thomas.markowski@pharmazie.uni-halle.de (T.M.); bodo.dobner@pharmazie.uni-halle.de (B.D.); 2 Institute of Pharmacy—Biophysical Pharmacy, MLU Halle-Wittenberg, Wolfgang-Langenbeck-Strasse 4, 06120 Halle (Saale), Germany; sindy.lindner@pharmazie.uni-halle.de; 3 Institute of Chemistry—Biophysical Chemistry, MLU Halle-Wittenberg, von-Danckelmann-Platz 4, 06120 Halle (Saale), Germany; annette.meister@chemie.uni-halle.de (A.M.); alfred.blume@chemie.uni-halle.de (A.B.); 4 Institute of Biochemistry and Biotechnology, MLU Halle-Wittenberg, Kurt-Mothes-Strasse 3, 06120 Halle (Saale), Germany

**Keywords:** bolaamphiphile, bolalipid, aggregation behavior, lipids, mixing behavior, pH, membranes

## Abstract

Investigations regarding the self-assembly of (bola)phospholipids in aqueous media are crucial to understand the complex relationship between chemical structure of lipids and the shape and size of their aggregates in water. Here, we introduce a new asymmetrical glycerol diether bolaphospholipid, the compound **Me_2_PE-Gly(2C16)C32-OH**. This bolalipid contains a long (C32) ω-hydroxy alkyl chain bond to glycerol in the *sn*-3 position, a C16 alkyl chain at the *sn*-2 position, and a protonable phosphodimethylethanolamine (Me_2_PE) headgroup at the *sn*-1 position of the glycerol. The aggregation behavior of this bolalipid was studied as a function of temperature and pH using transmission electron microscopy (TEM), differential scanning calorimetry (DSC), and Fourier transform infrared (FTIR) spectroscopy. We show that this bolalipid aggregates into condensed lamellar sheets in acidic milieu and in large sheet-like aggregates at neutral pH-value. By contrast, at a pH-value of 10, where the Me_2_PE headgroup is only partially protonated, small lipid disks with diameter 50–100 nm were additionally found. Moreover, the miscibility of this asymmetrical bolalipid with the bilayer-forming phosphatidylcholine DPPC was investigated by means of DSC and TEM. The incorporation of bolalipids into phospholipid membranes could result in stabilized liposomes applicable for drug delivery purposes. We show that mixtures of DPPC and **Me_2_PE-Gly(2C16)C32-OH** form large lamellar aggregates at pH of 5, 7, and 10. However, closed lipid vesicles (liposomes) with an increased thermal stability were not found.

## 1. Introduction

**Figure 1:**
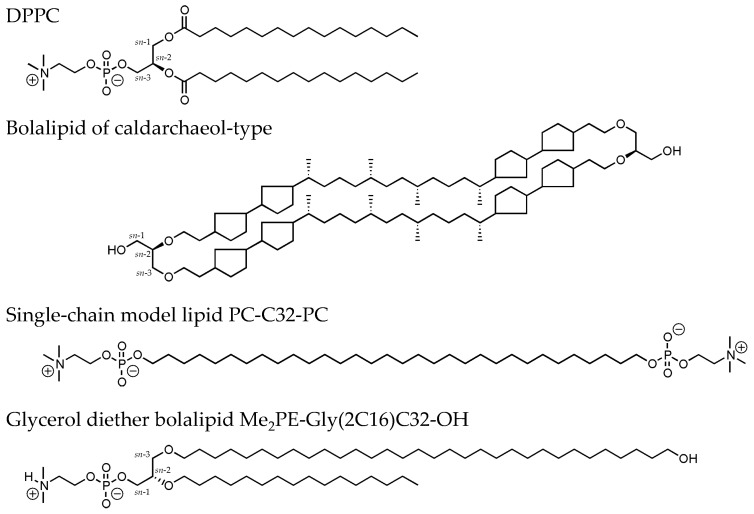
Chemical structure of 1,2-dipalmitoyl-*sn*-glycero-3-phosphocholine (DPPC), a natural occurring bolalipid of the caldarchaeol-type, the single-chain model lipid **PC-C32-PC**, and the new glycerol diether bolalipid **Me_2_PE-Gly(2C16)C32-OH** investigated in this work.

Bolalipids, which are also named bolaamphiphiles, are a special class of lipids. These bipolar lipids are composed of a long hydrophobic spacer—either a single or two alkyl chains connected by a glycerol moiety—and two polar headgroups attached to both ends [1]. These bipolar lipids can be found in the membranes of certain species of archaea, e.g., thermoacidophiles, where they are responsible for the outstanding stability of archaea against harsh living conditions, such as low pH values and/or high temperatures [2,3,4]. Reasons for this stability can be found in the chemical structure of archaeal bolalipids, which differs from the structure of membrane lipids of eukaryotes and bacteria, e.g., 1,2-dipalmitoyl-*sn*-glycero-3-phosphocholine (DPPC, Figure 1) [5,6,7]. Archaeal lipids are composed of fatty alcohols bound via ether bonds to the glycerol backbone in the invers *sn*-2,3 configuration and these alcohols contain isoprenoid methyl branches and/or a variable number of cyclopentane rings [2,3] (see chemical structure of a caldarchaeol-type bolalipid in Figure 1). With respect to the stability of archaeal membranes against external stress, such high temperatures, low pH-values, or hydrolyzing enzymes, the use of archaeal membrane lipids in material sciences, biotechnology, and pharmacy is very attractive [8,9,10,11,12,13]. Since these bipolar lipids are able to incorporate into bilayers composed of classical monopolar phospholipids, they can be used to stabilize liposomes for drug delivery applications. This approach was already tested for a wide range of artificial and natural bipolar lipids [10,14,15,16,17,18,19,20,21]. 

However, the main problem is obtaining sufficient amounts of the bolalipid material. Since the isolation of archaeal lipids from natural sources is very expensive and often leads to bolalipid mixtures, synthetic approaches were developed within the last decades. Kakinuma and co-workers published the total synthesis of naturally occurring archaeal membrane lipids [22,23,24,25], which is highly elaborate and, hence, not applicable for the production of bolalipids in larger scale. A more elegant way is to imitate the properties of these natural bolalipids by preparing simpler model compounds. Several groups have used this strategy of simplification [20,26,27,28,29]. In our group, we have also synthesized bola model lipids, for example, diglycerol tetraetherlipids that are composed of one membrane-spanning C32 alkyl chain and two shorter C16 alkyl chains bound to the glycerol backbone [30,31]. These tetraether lipids self-assemble into vesicular structures, and their thermal behavior is comparable to naturally occurring bolalipids. If the chemical structure of bolalipids is even more simplified, e.g., by omitting the glycerol moieties and the second short alkyl chains, a single-chain bolalipid consisting of a long C32 alkyl chain and two phosphocholine (PC) headgroups attached at both ends, the **PC-C32-PC** (Figure 1), is obtained [32,33]. This very simple archaeal model lipid self-assembles in water into long nanofibers and micellar aggregates in dependence of the temperature [32]; vesicular structures were not observed. This unusual aggregation behavior is traced back to the chemical structure of **PC-C32-PC**: If this bolalipid would be arranged in a monolayer with stretched bola molecules, void volume will be created between the bola molecules due to size differences between the cross-sections of the large PC headgroups and the smaller single alkyl chain. Hence, the aggregation in lamellar structures is energetically unfavorable and the self-assembly into fibrous aggregates is preferred. Within these nanofibers, the **PC-C32-PC** molecules are arranged side by side but slightly twisted relative to each other because of the bulky PC headgroups. This twisting finally leads to a helical superstructure of the fibers, which was previously confirmed by several optical and computational techniques [34,35]. 

Lastly, we introduced a novel class of bipolar lipids, the glycerol diether bolalipids [36]. This class of lipids is characterized by a glycerol backbone carrying a long C32 alkyl chain in the *sn*-3 position, a short C16 chain in the *sn*-2 position, and a PC headgroup in the *sn*-1 position. The membrane-spanning C32 chain further contains a hydroxy moiety at the end, which finally leads to a conical shape of the lipid molecule. We could show that this type of asymmetrical bolalipid self-assembles into large, sheet-like structures of high crystallinity [36]. Within these lamellar aggregates, the bola molecules are arranged in a fully interdigitated fashion. 

Now, we will present another glycerol diether bolalipid, which carries a phosphodimethyl-ethanolamine (Me_2_PE) instead of a PC headgroup: [2-*O*-hexadecyl-3-*O*-(32-hydroxydotriacontyl)-*sn*-glycer-1-yl]-2-[(dimethylammonio)ethyl phosphate], **Me_2_PE-Gly(2C16)C32-OH** (Figure 1). Due to the pH-dependent protonation state of this type of headgroup, a pH-dependent aggregation behavior of this bolalipid could be expected. A comparable situation was found for the symmetrical, single-chain **Me_2_PE-C32-Me_2_PE** [37,38] and also for asymmetrical bolalipids [39]. Here, we present the synthesis of **Me_2_PE-Gly(2C16)C32-OH** and physicochemical studies on the aggregation behavior of this bolalipid in dependence of temperature and pH value by means of transmission electron microscopy (TEM), differential scanning calorimetry (DSC), and Fourier transform infrared (FTIR) spectroscopy. Results from these investigations could be helpful to understand the complex relationship between chemical structure of bolalipids and their assemblies built in aqueous suspensions. Moreover, the mixing behavior of **Me_2_PE-Gly(2C16)C32-OH** with the saturated phosphatidylcholine DPPC was investigated by means of TEM and DSC. In previous studies, we could show that the symmetrical single-chain **PC-C32-PC** cannot be used as stabilizer of phospholipid bilayers [40]. The reason for that can be found in packing problems caused by a mismatch between the small cross-section of the single alkyl chain and the larger space requirement of the PC headgroup of **PC-C32-PC**. If the **PC-C32-PC** is inserted in a stretched manner into a phospholipid bilayer, void volume will be produced, which can be filled by neither phospholipid nor bolalipid. Consequently, the separation into bolalipid nanofibers and phospholipid vesicles is preferred [40]. With the introduction of a glycerol moiety as well as the second shorter alkyl chain on the one hand and the small hydroxy group at the end of the long C32 alkyl chain of **Me_2_PE-Gly(2C16)C32-OH** on the other hand, a better miscibility of bolalipid and phospholipid could be assumed. 

## 2. Materials and Methods 

### 2.1. Syntheses 

Chemicals for the synthesis were purchased from Sigma Aldrich Co. (Steinheim, Germany) and were used without further purification. 2-Bromoethylphosphoric acid dichloride was prepared according to the literature [41]. All solvents for synthetic purpose were dried and distilled before use. The purity of all compounds was checked by thin-layer chromatography (TLC) using silica gel 60 F254 plates (Merck, Darmstadt, Germany). The chromatograms were developed by means of bromothymol blue. Silica gel (Merck, 0.063–0.200 mm) was used for column chromatography of the products. Melting points were determined with Boetius apparatus. Optical rotation was quantified on a Polartronic E (Schmidt und Heansch) and [*α*]_D_ values are given in 10^−1^ deg cm^2^ g^−1^. ^1^H and ^13^C NMR spectra were recorded on a Agilent Technologies 400 MHz VNMRS spectrometer (Santa Clara, CA, USA) with the use of CDCl_3_ or CD_3_OD as internal standard. Chemical shifts (*δ*) are reported in parts per million (ppm). The coupling constants (*J*) are reported in Hz. Mass spectrometric data were obtained with a Finnigan LCQ-Classic (ESI–MS) (Thermo Seperation Products, San José, CA, USA). High-resolution mass spectra (HRMS) were recorded on a Thermo Fisher Scientific LTQ-Orbitrap mass spectrometer (Thermo Fisher Scientific Co., Waltham, MA, USA) with static nano-electrospray ionization. Copies of MS data (Figures S1, S2 and S5) and NMR spectra (Figures S3, S4, S6 and S7) can be found in the Supplementary Materials. 

#### 2.1.1. Preparation of **Me_2_PE-Gly(2C16)C32-OBn**

For the synthesis of the benzyl-blocked bolalipid **Me_2_PE-Gly(2C16)C32-OBn** a phosphorylation reaction of the precursor **HO-Gly(2C16)C32-OBn** [36] (1 equiv.; 0.21 mmol, 180 mg) with 2-Bromoethylphosphoric acid dichloride [42] (4 equiv.) and a subsequent quarternisation with an ethanolic solution of dimethylamine (10 equiv.) was used as described previously in detail [43]. The crude bolalipid was purified by column chromatography using CHCl_3_/MeOH/H_2_O as eluent. 

{3-*O*-[32-(Benzyloxy)dotriacontyl]-2-*O*-hexadecyl-*sn*-glycer-1-yl}-2-[(dimethylammonio)ethyl phosphate] (**Me_2_PE-Gly(2C16)C32-OBn**) was obtained (135 mg, 63%) as white solid. *R_f_* = 0.30 (CHCl_3_/MeOH/NH_3_, 40/10/1, *v*/*v*/*v*). *Mp* = 73–75 °C. ${\left[\alpha \right]}_{\text{D}}^{22}$ = +1.3 (*c* = 16 mg mL^−1^, CHCl_3_/MeOH, 1/1, *v*/*v*). ESI-MS: *m*/*z*: 1021.0 [M − H]^−^, 1023.1 [M + H]^+^, 1044.8 [M + Na]^+^. ^1^H NMR (400 MHz, CDCl_3_): *δ* = 0.85 (t, ^3^*J* = 6.6 Hz, 3H, –C***H***_3_), 1.08–1.35 (m, 82H, –(C***H***_2_)_28_–, –(C***H***_2_)_13_CH_3_), 1.50–1.55 (m, 4H, 2× –CH_2_C***H***_2_CH_2_O−), 1.57–1.60 (m, 2H, –CH_2_C***H***_2_CH_2_O–), 2.93 (s, 6H, –N(C***H***_3_)_2_), 3.37–3.57 (m, 11H, 4× –C***H***_2_O–, –C***H***O–, –NC***H***_2_CH_2_O–), 3.86–3.95 (m, 2H, –POC***H***_2_CH–), 4.27–4.30 (m, 2H, –NCH_2_C***H***_2_O–), 4.47 (s, 2H, –C***H***_2_C_6_H_5_), 7.24–7.31 ppm (m, 5H, –C_6_***H***_5_). ^13^C NMR (100 MHz, CDCl_3_): *δ* = 14.25 (–***C***H_3_), 22.83 (–***C***H_2_CH_3_), 26.30 (–OCH_2_CH_2_***C***H_2_–), 26.32 (–OCH_2_CH_2_***C***H_2_–), 26.35 (–OCH_2_CH_2_***C***H_2_–), 29.50–29.88 (–***C***H_2_–), 30.38 (–***C***H_2_–), 32.07 (–***C***H_2_CH_2_CH_3_), 43.92 (–N(***C***H_3_)_2_), 58.05 (d, *J*_C,P_ = 5.7 Hz, –NCH_2_***C***H_2_O–), 59.90 (–N***C***H_2_CH_2_O–), 65.90 (–PO***C***H_2_CH–), 70.43 (–***C***H_2_O–), 70.60 (–***C***H_2_O–), 70.63 (–***C***H_2_O–), 71.86 (–***C***H_2_O–), 72.91 (–***C***H_2_O–), 77.86 (d, *J*_C,P_ = 7.6 Hz, –***C***HO–), 127.38 (–***C***_6_H_5_, C4), 127.55 (–***C***_6_H_5_, C2,6), 128.27 (–***C***_6_H_5_, C3,5), 138.74 ppm (–***C***_6_H_5_, C1). HRMS: *m*/*z* calcd for C_62_H_121_O_7_NP [M + H]^+^ 1022.8875, found 1022.8927. 

#### 2.1.2. Preparation of **Me_2_PE-Gly(2C16)C32-OH**

The final bolalipid was prepared by hydrogenation of **Me_2_PE-Gly(2C16)C32-OBn** following a procedure described previously [36]. In brief, **Me_2_PE-Gly(2C16)C32-OBn** (0.071 mmol, 72 mg) and Pd (10 mg; 10%, on carbon) were dissolved in dry EtOH (20 mL). The mixture was stirred under hydrogen (5 atm) at 50 °C for 1 h. Then, the catalyst was removed by filtration, washed with CHCl_3_ several times, and the combined organic solutions were evaporated. The crude bolalipid was finally purified by column chromatography using CHCl_3_/MeOH/H_2_O as eluent and the gradient technique. 

[2-*O*-Hexadecyl-3-*O*-(32-hydroxydotriacontyl)-*sn*-glycer-1-yl]-2-[(dimethylammonio)ethyl phosphate] (**Me_2_PE-Gly(2C16)C32-OH**) was obtained (50 mg, 76%) as white crystalline solid. *R_f_* = 0.18 (CHCl_3_/MeOH/NH_3_, 40/10/1, *v*/*v*/*v*). *Mp* = 100–101 °C. ${\left[\alpha \right]}_{\text{D}}^{22}$ = −3.4 (*c* = 6 mg mL^−1^, CHCl_3_/MeOH, 1/1, *v*/*v*). ESI-MS: *m*/*z*: 932.7 [M + H]^+^, 954.6 [M + Na]^+^, 1864.6 [2M + H]^+^. ^1^H NMR (400 MHz, CDCl_3_): *δ* = 0.84 (t, ^3^*J* = 6.7 Hz, 3H, –C***H***_3_), 1.19–1.35 (m, 82H, –(C***H***_2_)_28_–, –(C***H***_2_)_13_CH_3_), 1.48–1.57 (m, 6H, 3× –CH_2_C***H***_2_CH_2_O–, H_2_O), 2.84 (s, 6H, –N(C***H***_3_)_2_), 3.18–3.22 (m, 2H, –NC***H***_2_CH_2_O–), 3.38–3.61 (m, 9H, 4× –C***H***_2_O–, –C***H***O–,), 3.90–3.94 (m, 2H, –POC***H***_2_CH–), 4.15–4.20 ppm (m, 2H, –NCH_2_C***H***_2_O–). ^13^C NMR (100 MHz, CDCl_3_/CD_3_OD): *δ* = 13.79 (–***C***H_3_), 22.48 (–***C***H_2_CH_3_), 25.61 (–OCH_2_CH_2_***C***H_2_–), 25.88 (–OCH_2_CH_2_***C***H_2_–), 25.92 (–OCH_2_CH_2_***C***H_2_–), 29.16–29.87 (–***C***H_2_–), 31.73 (HOCH_2_***C***H_2_–), 32.07 (–***C***H_2_CH_2_CH_3_), 43.00 (–N(***C***H_3_)_2_), 58.84 (–NCH_2_***C***H_2_O–), 62.20 (HO***C***H_2_–), 70.09 (–***C***H_2_O–), 70.42 (–***C***H_2_O–), 71.56 (–***C***H_2_O–), 77.20 ppm (–***C***HO–); signals for –N***C***H_2_CH_2_O– and –PO***C***H_2_CH– are below the detection limit due to poor solubility of the bolalipid in chloroform/methanol. HRMS: *m*/*z* calcd for C_55_H_114_O_7_NPNa [M + Na]^+^ 954.8225, found 954.8255. 

### 2.2. Physicochemical Methods 

#### 2.2.1. Chemicals 

1,2-Dipalmitoyl-*sn*-glycero-3-phosphocholine (DPPC) was purchased from Lipoid KG (Ludwigshafen, Germany). 

#### 2.2.2. Sample Preparation 

In a similar manner to a procedure from [44,45], an appropriate amount of **Me_2_PE-Gly(2C16)C32-OH** was suspended in acetate buffer (*c* = 10 mM, pH = 5.0), phosphate buffer (*c* = 10 mM, pH = 7.7), or carbonate buffer (*c* = 10 mM, pH = 10.0). For DSC measurements at pH = 10, 11, and 12, carbonate buffer was firstly used to dissolve the bolalipid and the final pH was adjusted with conc. NaOH. Homogeneous suspensions were obtained by heating and vortexing. Binary lipid mixtures were prepared from lipid stock solutions in CHCl_3_/MeOH (2/1, *v*/*v*) as solvent by mixing appropriate volumes of the stock solutions. The organic solvent was then removed in a stream of N_2_ and the resulting lipid films were kept in an evacuated flask for 24 h at room temperature to remove residual traces of the solvent. The suspensions were then prepared by adding a certain volume of the appropriate buffer solution (see above) to obtain a total lipid concentration of 3 mM. The samples were heated to 90 °C and vigorously vortexed to obtain an almost homogeneous suspension. 

#### 2.2.3. Differential Scanning Calorimetry (DSC) 

DSC measurements were performed using a MicroCal VP-DSC differential scanning calorimeter (MicroCal Inc., Northampton, MA, USA). Before the measurements, the sample suspension (*c* = 1 mg mL^−1^ for the pure bolalipid, *c* = 3 mM for lipid mixtures) and the buffer reference were degassed under vacuum while stirring. A heating rate of 60 K h^−1^ was used, and the measurements were performed in the temperature interval from 5 °C to 95 °C. To check the reproducibility, three consecutive scans were recorded for each sample. The buffer/buffer baseline was subtracted from the thermogram of the sample, and the DSC scans were evaluated using MicroCal Origin 8.0 software (MicroCal Inc., Northampton, MA, USA). 

#### 2.2.4. Fourier Transform Infrared (FTIR) Spectroscopy 

Infrared spectra were obtained on a Bruker Vector 22 (Bruker Optik GmbH, Karlsruhe, Germany) Fourier transform spectrometer with DTGS (deuterated-triglycine sulfate) detector operation at 2 cm^−1^ resolution. The bolalipid suspension (*c* = 200 mg mL^−1^ in acetate buffer at pH 5) was placed between two CaF_2_ windows, separated by a 6 µm teflon spacer. IR spectra were recorded in steps of 2 K in the temperature range from 9 to 89 °C. The temperature was adjusted with a Haake F6 thermostat (C25, Thermo Electron Corporation, Karlsruhe, Germany) and controlled with Delphi-based, home-written software. After an equilibration time of 8 min, 64 scans were recorded and accumulated. The corresponding solvent spectra (acetate buffer) were subtracted from the sample spectra using the OPUS software supplied by Bruker. 

#### 2.2.5. Transmission Electron Microscopy (TEM) 

The samples were prepared by spreading 5 µL of the bolalipid suspension (*c* = 0.05 mg mL^−1^ in case of the pure bolalipid, *c* = 60 µM in case of lipid mixtures) onto a copper grid coated with a Formvar film. After 1 min, excess liquid was blotted off with filter paper and 5 μL of 1% aqueous uranyl acetate solution were placed onto the grid and drained off after 1 min. All specimens were examined after drying with a Zeiss EM 900 transmission electron microscope (Carl Zeiss Microscopy GmbH, Jena, Germany). 

## 3. Results and Discussion 

### 3.1. Synthesis of **Me_2_PE-Gly(2C16)C32-OH**

**Scheme 1:**
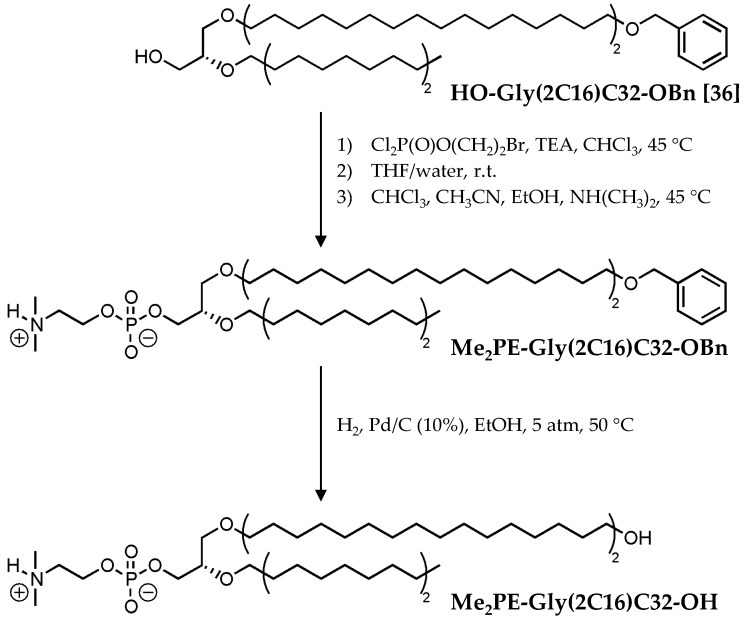
Synthesis of **Me_2_PE-Gly(2C16)C32-OH**; TEA: triethylamine; r.t.: room temperature.

The preparation of the new glycerol diether bolalipid bearing a protonable phosphodimethyl-ethanolamine (Me_2_PE) headgroup, the [2-*O*-hexadecyl-3-*O*-(32-hydroxydotriacontyl)-*sn*-glycer-1-yl]-2-[(dimethylammonio)ethyl phosphate] (**Me_2_PE-Gly(2C16)C32-OH**), is based on the synthesis of the phosphocholine (PC) analog published previously [36]. In brief, the introduction of the Me_2_PE headgroup into the precursor compound **HO-Gly(2C16)C32-OBn** was carried out by the classical phosphorylation reaction described by Eibl et al. [42] using the 2-bromoethylphosphoric acid dichloride [41] as phosphorylating reagent followed by quarternisation with dimethylamine in CHCl_3_/CH_3_CN/EtOH. After purification of **Me_2_PE-Gly(2C16)C32-OBn**, which was obtained in 63% yield, the remaining benzyl blocking group was removed using a hydrogenation reaction on palladium/carbon (10%) in EtOH at 5 bar and 50 °C, and the new asymmetrical glycerol diether bolalipid **Me_2_PE-Gly(2C16)C32-OH** was obtained in 76% yield after purification (Scheme 1). 

### 3.2. Temperature-Dependent Aggregation Behavior of **Me_2_PE-Gly(2C16)C32-OH**

As a first observation, **Me_2_PE-Gly(2C16)C32-OH** is hardly dispersible in acetate buffer at pH 5, where the Me_2_PE headgroup is protonated and, hence, in its zwitterionic state. To get a homogeneous, opalescent suspension, 25% (*v*/*v*) ethanol was added and the mixture was treated with repeated cycles of heating and vortexing. An attempt to prepare liposomes of the bolalipid suspension with the use of sonication and/or extrusion through a polycarbonate membrane of 100 nm pore size failed and dynamic light scattering (DLS) measurements of the bolalipid suspension right after extrusion revealed the presence of very large particles with high polydispersity (data not shown). This indicates that **Me_2_PE-Gly(2C16)C32-OH** probably self-assembles into very large, sheet-like aggregates rather than in vesicular structures or other smaller aggregates such as micelles. 

**Figure 2:**
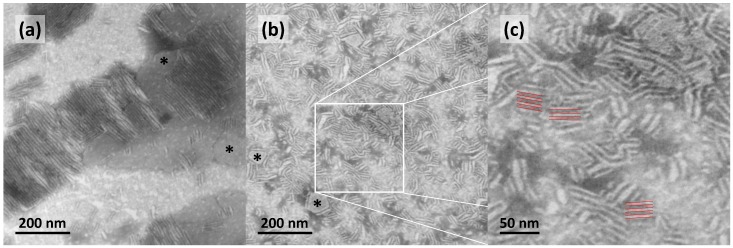
(**a**–**c**) Transmission electron microscopy (TEM) images of aqueous suspensions (*c* = 0.03 mg mL^−1^ in acetate buffer at pH = 5) of the bolalipid **Me_2_PE-Gly(2C16)C32-OH** at different magnifications; the inset in (**b**) is depicted in (**c**); the distance between layers is about 5 to 6 nm. The black asterisks in (**a**,**b**) point to lamellar sheets oriented parallel to the grid surface. The samples were prepared at about 22 °C and stained with uranyl acetate.

To visualize the aggregates of **Me_2_PE-Gly(2C16)C32-OH** in aqueous suspension, TEM images from negatively stained samples (*c* = 0.03 mg mL^−1^ in acetate buffer at pH = 5; prepared at 22 °C ) were obtained (see Figure 2). 

The TEM image of **Me_2_PE-Gly(2C16)C32-OH** in acetate buffer shows the formation of large patches of condensed lamellar sheets, which are either oriented parallel (see black asterisks in Figure 2a,b) or perpendicular to the grid surface. In some areas, the lamellar aggregates are oriented over a large distance in the same manner (Figure 2a), whereas, in other regions, the stacked lamellae are more or less randomly ordered (Figure 2b). A closer inspection of the layered structures revealed a distance between the layers of about 5 to 6 nm (see red lines in Figure 2c). Other aggregate structures, such as small, circular sheets as found for **PC-Gly(2C16)C32-OH** [36], or liposomes were not observed. 

**Figure 3:**
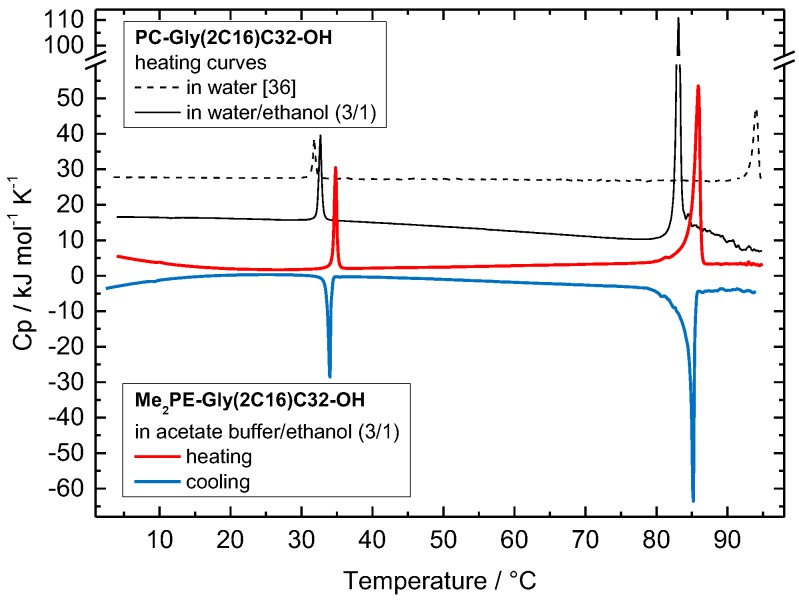
Differential scanning calorimetry (DSC) heating (red solid line) and cooling (blue solid line) curve of a **Me_2_PE-Gly(2C16)C32-OH** suspension (*c* = 1 mg mL^−1^) in a mixture of acetate buffer (pH = 5.0, 10 mM) and ethanol (3/1, *v*/*v*). The DSC heating curves of **PC-Gly(2C16)C32-OH** either in water/ethanol (3/1, *v*/*v*; black solid line) or in water (black dashed line) are shown for comparison. The heating rate was 60 K h^−1^. The curves are shifted vertically for clarity.

As mentioned before, 25% of ethanol was added to the **Me_2_PE-Gly(2C16)C32-OH** suspension in acetate buffer (pH = 5.0, 10 mM) to get a homogeneous opaque dispersion. The DSC heating scan of this suspension shows two endothermic and very cooperative transitions at *T*_1_ = 34.8 °C (fwhm = 0.41 K) and at *T*_2_ = 85.9 °C (fwhm = 0.85 K), the latter transition includes a small shoulder at 81.4 °C (Figure 3, red solid line). The cooling curve depicts the same peak pattern with virtually no hysteresis: the high-temperature peak is observed at 85.2 °C (fwhm = 0.37 K) and the second peak at 34.0 °C (fwhm = 0.46 K; Figure 3, blue solid line). 

To compare these DSC data with the data of the PC counterpart studied previously [36], we have to re-investigate the **PC-Gly(2C16)C32-OH** in the new solvent, namely water/ethanol (3/1, *v*/*v*). The original DSC data of **PC-Gly(2C16)C32-OH** measured in pure water depict two endothermic transitions at 31.8 °C and 94.0 °C (Figure 3, black dashed line) [36]. With the change to water/ethanol (3/1, *v*/*v*; see black solid line in Figure 3), the first transition peak is slightly shifted to higher temperatures (*T* = 32.7 °C, Δ*T* = +0.9 K, fwhm = 0.45 K, Δ*H* = 12.7 kJ mol^−1^), whereas the second peak is distinctly shifted to lower temperatures (*T* = 83.1 °C, Δ*T* = −10.9 K, fwhm = 0.51 K, Δ*H* = 65 kJ mol^−1^). Since the high temperature transition of **PC-Gly(2C16)C32-OH** is connected to the melting of the alkyl chains, i.e., the transition from gel to liquid-crystalline phase [36], ethanol destabilizes the gel phase of **PC-Gly(2C16)C32-OH**. The comparison between the new **Me_2_PE-Gly(2C16)C32-OH** and the PC counterpart in analogous solvents reveals that the temperatures of both DSC transitions are slightly increased by about 2.1 and 2.8 K, respectively, for the Me_2_PE derivative. This increase in *T*_m_ is obviously due to the possibility of protonated Me_2_PE headgroups to form hydrogen bonds between bolalipid molecules, which stabilizes the aggregate structure of **Me_2_PE-Gly(2C16)C32-OH** molecules. However, this stabilizing effect of hydrogen bonds is not as pronounced as in DPPC compared to 1,2-dipalmitoyl-*sn*-glycero-3-phosphoethanolamine (DPPE) [46], which is obviously due to the existence of already densely packed alkyl chains in the **PC-Gly(2C16)C32-OH** aggregates [36]. 

**Figure 4:**
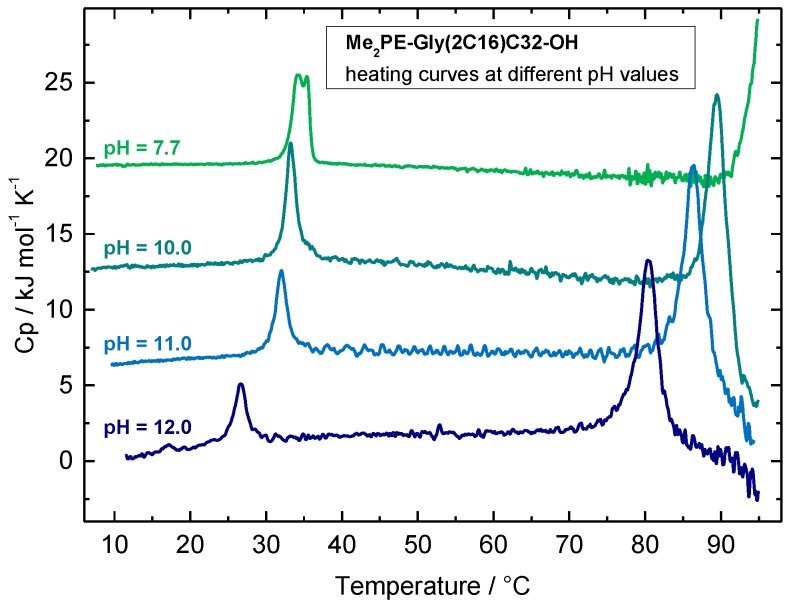
DSC heating curves of a **Me_2_PE-Gly(2C16)C32-OH** suspension (*c* = 1 mg mL^−1^) in variable buffer solutions at different pH values: phosphate buffer at pH = 7.7 (green line); carbonate buffer at pH = 10.0 (cyan line), pH = 11.0 (blue line), and pH = 12 (dark blue line). The heating rate was 60 K h^−1^. The curves are shifted vertically for clarity.

Since the charge of the headgroup of **Me_2_PE-Gly(2C16)C32-OH** depends on the pH-value (the p*K*_a_ values of the Me_2_PE group are 0.98 ± 0.50 for p*K*_a_1 and 8.46 ± 0.28 for p*K*_a_2, ACD/pKa software), the question arose how different pH values influence the aggregation behavior. As previously shown for the symmetrical, single-chain **Me_2_PE-C32-Me_2_PE**, an increase in pH led to a decrease in the transition temperature due to negatively charged and, hence, repulsive headgroups [37,38,47]. The DSC heating curves of **Me_2_PE-Gly(2C16)C32-OH** at different pH values are shown in Figure 4. At pH = 7.7 (phosphate buffer), the DSC curve reveals a first transition peak with two maxima at 34.2 and 35.4 °C, respectively, and the beginning of a second transition at about 91 °C (Figure 4, green line). However, these *T*_m_ values are not directly comparable to both *T*_m_ values obtained at pH = 5.0 (Figure 3), because, in the latter case, 25% ethanol has to be added to the acetate buffer to ensure a homogeneous dispersion of the bolalipid, which was not necessary for the dispersions at pH = 7.7. However, taking the effect of ethanol into account (see above), both transition temperatures are more or less comparable. 

If the pH value is further increased, both transitions observed in DSC are gradually shifted to lower temperatures, finally reaching *T*_1_ = 26.6 °C and *T*_2_ = 80.4 °C, respectively, at pH = 12 (Figure 4, dark blue line). Complete data of DSC measurements from **Me_2_PE-Gly(2C16)C32-OH** in different buffer systems are summarized in Table 1. The increased noise in the DSC scans shown in Figure 4, especially between the first and the second transition, is probably due to very small amounts of a precipitate that occurs inside the measuring cell with increasing temperature. 

The shift in *T*_m_ values is most pronounced between pH 11 and 12, indicating that the apparent p*K*_a_ of the Me_2_PE headgroup within the **Me_2_PE-Gly(2C16)C32-OH** aggregates is within this pH region. However, the effect of deprotonation on *T*_m_ is not as pronounced as it was for the symmetrical **Me_2_PE-C32-Me_2_PE** [38,47]. The reasons for that could be the following:

- There is only one Me_2_PE headgroup present in **Me_2_PE-Gly(2C16)C32-OH** compared to two headgroups in **Me_2_PE-C32-Me_2_PE**, whereas the overall size (molar mass) of the asymmetrical **Me_2_PE-Gly(2C16)C32-OH** is larger with respect to the symmetrical **Me_2_PE-C32-Me_2_PE**.
- If we assume an interdigitated orientation of the **Me_2_PE-Gly(2C16)C32-OH** molecules within the aggregates, as was found for the PC analog [36], the terminal hydroxy group might compensate the negative charge of the deprotonated Me_2_PE headgroup.
- For the pH-dependent experiments using the symmetrical **Me_2_PE-C32-Me_2_PE** [38], unbuffered suspensions were used and the pH was adjusted with conc. NaOH solution. Here, a carbonate buffer was used. The additional ions present in the solution (sodium and potassium) could partially shield the negative charge of the deprotonated Me_2_PE headgroup.

**Figure 5:**
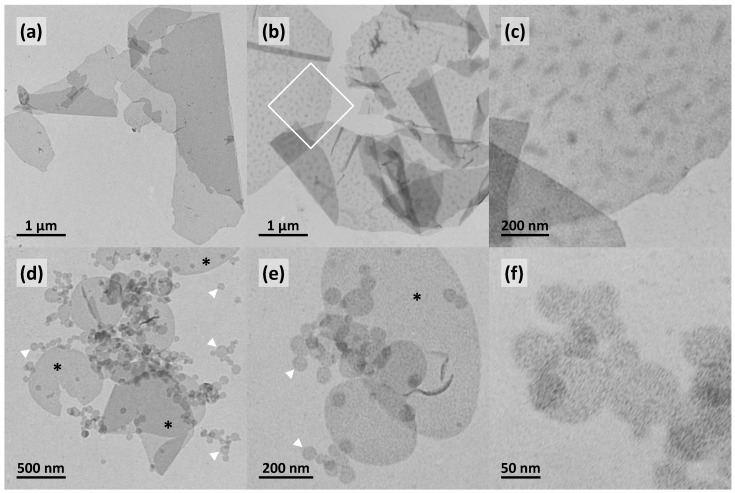
TEM images of aqueous suspensions (*c* = 0.05 mg mL^−1^) of **Me_2_PE-Gly(2C16)C32-OH**: in phosphate buffer at pH = 7.7 (**a**–**c**); and carbonate buffer at pH = 10.0 (**d**–**f**), at different magnifications; the inset in (**b**) is depicted in (**c**). The black asterisks in (**d**,**e**) point to larger lamellar sheets, whereas the white arrowheads point to smaller, round, disk-like objects. Samples were prepared at 22 °C and stained with uranyl acetate.

To further clarify the influence of the pH on the aggregation behavior of **Me_2_PE-Gly(2C16)C32-OH**, TEM images from negatively stained samples (*c* = 0.05 mg mL^−1^, in phosphate or carbonate buffer) were obtained. At pH = 7.7, TEM images of **Me_2_PE-Gly(2C16)C32-OH** shows the formation of very large lamellar aggregates (Figure 5a) of several micrometers, which are sometimes folded due to the drying process during the sample preparation. A characteristic stacking of lamellar sheets, as found for **Me_2_PE-Gly(2C16)C32-OH** in acetate buffer at pH = 5 (see Figure 2), was not observed. Other types of structures, such as crushed vesicles or smaller disk-like assemblies, were also not found. In some cases, the lamellar aggregates reveal the presence of darker, randomly arranged patches (see Figure 5b and the magnification in Figure 5c). The origin of these patches is not understood up to now. With the change to pH = 10, the self-assembly of **Me_2_PE-Gly(2C16)C32-OH** changes slightly. TEM images from a sample suspension under alkaline conditions depicts two types of aggregates: One the one hand, large lamellar aggregates of maximum 800 nm in size are found (black asterisks in Figure 5d,e), which are comparable to structures found at pH = 7.7 (see above), albeit not as large as those. On the other hand, very small and rounded aggregates are observed (see white arrowheads in Figure 5d,e). These disk-like aggregates have diameter 50–100 nm and show no additional structuring. Up to now, we can only speculate about the origin of these small disks, but it seems conceivable that negatively charged **Me_2_PE-Gly(2C16)C32-OH** molecules are separated from the zwitterionic ones and accumulate at the rim of the aggregates, further preventing the fusion of disks into larger assemblies. Additional investigations using dynamic light scattering and/or zeta potential measurements are mandatory to prove or refute this assumption. 

Temperature-dependent FTIR experiments can provide information about the nature of thermotropic transitions observed in the DSC. For example, the position of the symmetric [*ν*_s_(CH_2_)] and antisymmetric [*ν*_as_(CH_2_)] methylene stretching vibrational band tells us something about the conformational order of the alkyl chain, i.e., whether the chains are in an all-*trans* conformation and well-ordered or in the fluid state [48,49]. Additionally, the position of the methylene scissoring vibrational band [*δ*(CH_2_)] can be analyzed, which gives information about the alkyl chain packing mode, whether hexagonal, orthorhombic, triclinic, or other phases are formed [49]. 

**Figure 6:**
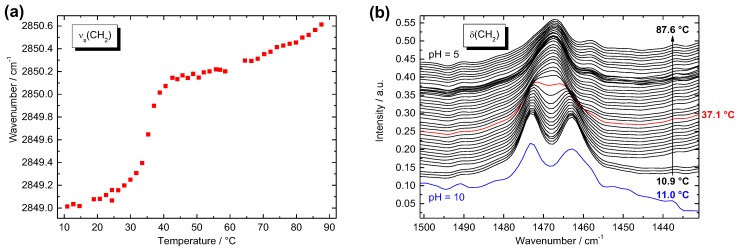
Fourier transform infrared (FTIR) spectroscopy data of **Me_2_PE-Gly(2C16)C32-OH** (*c* = 100 mg mL^−1^ in acetate buffer, pH = 5, 300 mM): (**a**) wavenumber of the symmetric methylene stretching vibrational band as a function of temperature; and (**b**) FTIR spectra at different temperatures in the region of the methylene scissoring vibrational band; in black: heating from 10.9 °C (bottom) to 87.6 °C (top) in acetate buffer at pH = 5.0 with increments of 2 K (the red curve at 37.1 °C indicates a change in the band positions); in blue: FTIR spectrum in carbonate buffer at pH = 10 at 11.0 °C. The individual curves are shifted vertically for clarity.

For **Me_2_PE-Gly(2C16)C32-OH** (*c* = 100 mg mL^−1^ in acetate buffer at pH = 5), the frequency of the *ν*_s_(CH_2_) band at low temperatures is located at 2849.0 cm^−1^ (Figure 6a). This value is indicative for alkyl chains in all-*trans* conformation, i.e., the bolalipid is in the gel or sub-gel phase below *T*_1_. Upon heating, the wavenumber of this band increases to some extent at the beginning and shows pronounced jump between 32 and 41 °C, reaching a value of 2850.1 cm^−1^ at *T* = 42.6 °C. This jump, which is concomitant with the first endothermic transition observed in the DSC, indicates a slightly increased flexibility of the alkyl chains, although a wavenumber of around 2850 cm^−1^ for the *ν*_s_(CH_2_) band is still too low for a considerable amount of *gauche* conformers within the alkyl chain. Hence, the **Me_2_PE-Gly(2C16)C32-OH** is still in the gel phase above *T*_1_. If the temperature is further increased, the wavenumber of the *ν*_s_(CH_2_) band shows a second increase starting at *T* ≈ 70 °C, finally reaching 2850.6 cm^−1^ at *T* = 87.6 °C. At this temperature, the alkyl chains obviously start to “melt” and a liquid-crystalline phase is formed. 

For a closer look at the nature of the transitions, the temperature-dependent position of the *δ*(CH_2_) band was analyzed (Figure 6b). At low temperatures, a characteristic splitting of the CH_2_ deformation band (1473.0 and 1463.0 cm^−1^) is observed, which is indicative of a highly ordered, orthorhombic (quasi-crystalline) alkyl chain packing [49]. At a temperature of *T* = 37 °C, both bands merge to a single band (1467.4 cm^−1^; Figure 6b, red line) including a shoulder at higher wavenumbers, which disappears upon further heating. The frequency of the *δ*(CH_2_) band then remains virtually constant up to high temperatures. The position of this single CH_2_ deformation band is indicative of a hexagonal packing of alkyl chains. This means that the first endothermic transition is connected with a change in the chain packing mode from an ordered orthorhombic *L*_β′c_ (*G*_o_) to a—still ordered—hexagonal *L*_β′_ phase. However, the shoulder at higher wavenumbers above *T*_1_ could also be an indication for a change from a *G*_o_ phase to an ordinary orthorhombic *L*_β′_ (*G*_d_) phase, which was found previously for long-chain monopolar diacylphosphatidylcholines at very low temperatures [50] and also for the PC analog of **Me_2_PE-Gly(2C16)C32-OH** [36]. Under alkaline conditions (in carbonate buffer at pH = 10.0), the characteristic splitting of the *δ*(CH_2_) band is found again at temperatures below *T*_1_ (Figure 6b, blue line), indicating that the very dense packing of the alkyl chains in the low temperature phase is unaffected by a change in pH-values from 5 to 10. 

### 3.3. Mixing Behavior of **Me_2_PE-Gly(2C16)C32-OH** with DPPC 

To get an impression of the miscibility of our novel bolalipid with bilayer-forming phospholipids, **Me_2_PE-Gly(2C16)C32-OH** was mixed with DPPC in two different mixing ratios, namely 1:4 (bolalipid:DPPC, *n*:*n*) and 1:1. Additionally, both mixtures were investigated at three different pH-values to check whether different protonation states of **Me_2_PE-Gly(2C16)C32-OH** have an influence on the mixing behavior. In previous studies, we showed that single-chain bolalipids, such as **PC-C32-PC**, could not be incorporated into bilayers of DPPC, 1,2-dimyristoyl-*sn*-glycero-3-phosphocholine (DMPC), or 1-palmitoyl-2-oleoyl-*sn*-glycero-3-phosphocholine (POPC) [40]. By contrast, single-chain bolalipids bearing an alkyl chain modification, such as phenyl rings, methyl or acetylene groups, were partially miscible with DPPC. Closed vesicular structures were not observed; only bilayer fragments, small disk-like aggregates, and/or elongated micelles were formed instead [44,45,51,52]. 

**Figure 7:**
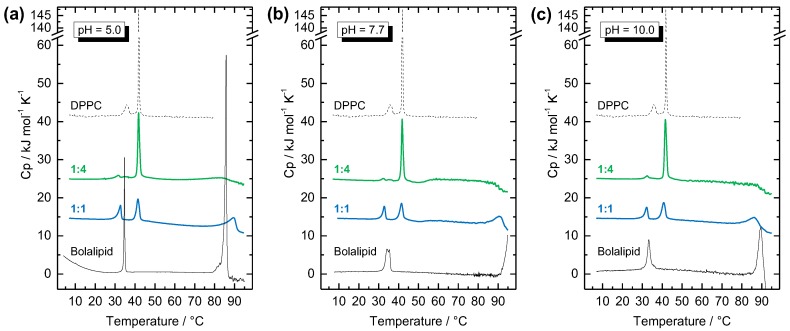
DSC heating curves of different **Me_2_PE-Gly(2C16)C32-OH**:DPPC mixtures (*c* = 3 mM) in: (**a**) acetate buffer at pH = 5.0; (**b**) phosphate buffer at pH = 7.7; and (**c**) carbonate buffer at pH = 10.0. The molar ratios are 1:4 (bolalipid:DPPC, *n*:*n*; green line) and 1:1 (blue line). DSC data of pure **Me_2_PE-Gly(2C16)C32-OH** (*c* = 1 mg mL^−1^, black solid line; in (**a**) with 25%, *v*/*v*, of ethanol) and DPPC (*c* = 1 mg mL^−1^, black dashed line) are shown for comparison. The heating rate was 60 K h^−1^ and the second heating scan is shown for all measurements. The curves are shifted vertically for clarity.

The mixing behavior of **Me_2_PE-Gly(2C16)C32-OH** with DPPC was studied by DSC and TEM. We firstly investigated the thermotropic behavior of **Me_2_PE-Gly(2C16)C32-OH**:DPPC mixtures (*c* = 3 mM) at different pH-values. The DSC heating scans are depicted in Figure 7. 

The DSC heating scan of the pure DPPC shows the two well-known endothermic transitions (see black dashed line in Figure 7): at *T*_p_ = 36.4 °C, the pre-transition from the *L*_β’_-phase to the ripple-phase (*P*_β′_); and, at *T*_m_ = 41.8 °C, the very cooperative main transition, where the alkyl chains became fluid and the *L*_α_-phase is formed. By adding different molar amounts of **Me_2_PE-Gly(2C16)C32-OH** to DPPC, two important findings can be deduced: First, by comparing the various pH-values used in the mixing experiments, i.e., acetate buffer at pH = 5.0 (Figure 7a), phosphate buffer at pH = 7.7 (Figure 7b), and carbonate buffer at pH = 10.0 (Figure 7c), no differences can be found. This phenomenon implies that either the apparent p*K*_a_-value of Me_2_PE headgroups is above 10—a fact already suggested from the DSC experiments of the pure **Me_2_PE-Gly(2C16)C32-OH** (see above)—or the pH-value of the suspension and, hence, the protonation state of the bolalipid has no influence on the mixing behavior. Second, the transition temperatures from **Me_2_PE-Gly(2C16)C32-OH** and DPPC, respectively, remain virtually unaffected in the two different mixed system. In the 1:4 (bolalipid: DPPC, *n*:*n*) mixture, i.e., 20 mol % of **Me_2_PE-Gly(2C16)C32-OH**, a main transition is observed at *T*_m_ of DPPC (Figure 7, green lines) and a small transition is found below *T*_1_ of the bolalipid. Additionally, a very broad transition for the suspension in acetate buffer at pH = 5.0 as well as a decrease in the *C*_p_-value for pH = 7.7 and pH = 10.0 can be found at high temperatures within the temperatures range of *T*_2_ of **Me_2_PE-Gly(2C16)C32-OH**. In equimolar mixtures (Figure 7, blue lines), three endothermic transitions can be found for all three different pH-values investigated: a first transition is again below *T*_1_ of the bolalipid, a second one is slightly below *T*_m_ of DPPC, and a third, broad transition is found between 85 and 95 °C, again in the temperature range of *T*_2_ of **Me_2_PE-Gly(2C16)C32-OH**. 

Since separated DSC transitions are found for each component in the DSC scans of the mixture, one can conclude that the bolalipid **Me_2_PE-Gly(2C16)C32-OH** and the saturated phosphatidylcholine DPPC shows almost no miscibility in the gel phase and that the bolalipid thus is not suitable to stabilize phospholipid liposomes. Moreover, since the second, high temperature transition (*T*_2_) of the bolalipid can be detected in the 1:1 mixture, even a miscibility in the fluid phase of DPPC is doubtful. DSC data for mixed systems are summarized in Table 2. 

**Figure 8:**
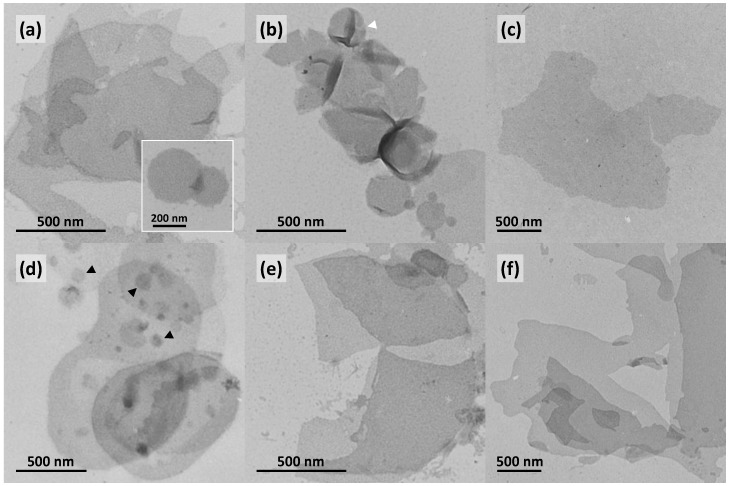
TEM images of aqueous suspensions (*c* = 0.05 mg mL^−1^) of **Me_2_PE-Gly(2C16)C32-OH**:DPPC mixtures at different molar ratios: 1:4 (**a**–**c**); and 1:1 (**d**–**f**); in acetate buffer at pH = 5.0 (**a**,**d**); phosphate buffer at pH = 7.7 (**b**,**e**); and carbonate buffer at pH = 10.0 (**c**,**f**), at different magnifications. The inset in (**a**) shows a small round aggregate. The white arrowhead in (**b**) points to a crushed vesicle, whereas the black arrowheads in (**d**) point to small aggregates. Samples were prepared at 22–24 °C and stained with uranyl acetate.

To get an idea about the aggregate structure of the **Me_2_PE-Gly(2C16)C32-OH**:DPPC systems at different pH-values, TEM images were obtained from negatively stained samples. All specimens were prepared at 22–24 °C, i.e., below the transitions observed in DSC. The images are shown in Figure 8. 

In almost every case, large sheet-like aggregates are observed in TEM images of **Me_2_PE-Gly(2C16)C32-OH**:DPPC mixtures (Figure 8). Smaller, round-shaped object are only found in mixtures at pH = 5.0 (see inset in Figure 8a and black arrowheads in Figure 8d). Liposomal structures, which appear as crushed vesicles with a characteristic folding in electron micrographs of negative stained samples, are only observed in the 1:4 mixture in phosphate buffer (see white arrowhead in Figure 8b). From the EM images we can conclude that **Me_2_PE-Gly(2C16)C32-OH** and DPPC should be partially miscible in such a way that both components are part of one large sheet. The opposite case was found for a mixture of DPPC with an asymmetric, single-chain bolalipid—here, DPPC vesicles and sheet-like aggregates of the bolalipid were observed concomitantly [51]. However, since DSC data indicate an immiscibility of **Me_2_PE-Gly(2C16)C32-OH** and DPPC on the molecular level, a segregation of both substances and, hence, the formation of lipid clusters within one aggregate cannot be excluded. To prove this assumption, further physicochemical studies on **Me_2_PE-Gly(2C16)C32-OH**:phospholipid mixtures are mandatory. 

## 4. Conclusions 

The stabilization of phospholipid bilayer membranes using bipolar lipids (bolalipids) is a promising approach in drug delivery applications. With respect to the limited excess of natural (archaeal) bolalipids and the time-consuming total synthesis of these complex lipids, the simplification of the chemical structure of bolalipids by maintaining their stabilizing properties is an elegant way to create new lipid compounds. Needless to say, the physicochemical behavior of these new substances in aqueous suspension as well as their miscibility with classical phospholipids has to be analyzed prior the application. 

With the synthesis of **Me_2_PE-Gly(2C16)C32-OH**, we continue our work on the characterization of a novel class of bolalipids, the glycerol diether lipids. Besides, the membrane-spanning C32 alkyl chain including a ω-hydroxy group and the short C16 alkyl chain, the new bolalipid bears a protonable phosphodimethylethanolamine (Me_2_PE) headgroup. Under acidic conditions, where the Me_2_PE headgroup is protonated and in its zwitterionic state, the **Me_2_PE-Gly(2C16)C32-OH** self-assembles into condensed lamellar sheets with densely packed alkyl chains. A similar aggregate structure with very high chain order at ambient temperature was previously observed for the PC analog of our bolalipid. By increasing the pH-value of the suspension, the aggregate form slightly changes to large lamellar sheets at pH = 7.7 and lamellar structures along with small rounded disks at pH = 10. However, the overall thermal behavior—a first endothermic transition at *T*_1_ = 27–35 °C from a crystalline (sub-gel) phase to a gel phase and a second transition at *T*_2_ > 80 °C from a gel to a liquid-crystalline phase—is unaffected by the pH-value of the suspension. Hence, the thermal behavior of **Me_2_PE-Gly(2C16)C32-OH** is again comparable to its PC analog described previously. This leads to the assumption that both the PC and the Me_2_PE headgroup behave quite similar under “physiological” conditions, i.e., pH-values between 5 and 10 and the presence of counter ions. Nevertheless, the protonated Me_2_PE headgroup is able to form hydrogen bonds, which stabilizes the structure of aggregates and leads to slightly higher transition temperatures. 

In the second part, we investigated the miscibility of the new glycerol diether bolalipid with one example of a bilayer-forming phospholipid, the diester phospholipid DPPC. An incorporation of the bolalipid in an extended conformation into a DPPC membrane could possibly result in a higher thermal stability of the membrane. However, we could demonstrate that **Me_2_PE-Gly(2C16)C32-OH** is not miscible with DPPC on a molecular level, independent from the pH-value used in the mixing experiments, as separated transitions could be detected from both mixing partners in the DSC scans. Since large lamellar aggregates are observed in electron micrographs of all mixed samples, a segregation of both lipid components within the aggregate is conceivable. We think that the high quasi-crystalline order of the **Me_2_PE-Gly(2C16)C32-OH** molecules is the lamellar structure induced by the long, stretched C32 alkyl chain prevents a complete mixing with classical phospholipids such as DPPC. Thus, the compound is not suited for the formation of stabilized liposomes applicable for drug delivery purposes. To achieve a better miscibility, we will insert modifications in the alkyl chains of the glycerol diether bolalipid, for example phytanyl or unsaturated chain, to create more disorder and we will also use unsaturated phospholipids in mixing studies. Moreover, we will consider how a second larger headgroup at the ω-position of the long alkyl chain influences the aggregation and mixing behavior with bilayer-forming phospholipids. All these aspects are part of ongoing research. 

## Figures and Tables

**Table 1:** DSC data of **Me_2_PE-Gly(2C16)C32-OH** suspensions (*c* = 1 mg mL^−1^) at various pH values using different buffer systems (*c* = 10 mM) as indicated; data are taken from the second heating scan (heating rate 60 K h^−1^).

pH value	Buffer system	First transition (*T*_1_)			Second transition (*T*_2_)		
		*T*/°C	Fwhm/K	Δ*H*/kJ mol^−1^	*T*/°C	Fwhm/K	Δ*H*/kJ mol^−1^
5.0	acetate buffer/ethanol (3/1, *v*/*v*)	34.8	0.4	13.3	85.9	0.9	60.2
7.7	phosphate buffer	34.2/35.4	2.4	15.2	91 ^1^	n.a.	n.a.
10.0	carbonate buffer	33.2	1.5	15.8	89.5	3.0	52.8
11.0	carbonate buffer + NaOH	32.0	1.7	11.0	86.4	2.8	44.1
12.0	carbonate buffer + NaOH	26.6	1.8	8.9	80.4	2.7	37.2

^1^ beginning of the transition, peak maximum not detectable; n.a. = not assignable.

**Table 2:** DSC data of **Me_2_PE-Gly(2C16)C32-OH**:DPPC mixtures (*c* = 3 mM) at various pH values using different buffer systems (*c* = 10 mM; acetate buffer at pH = 5.0, phosphate buffer at pH = 7.7, carbonate buffer at pH = 10.0); data are taken from the second heating scan (heating rate 60 K h^−1^).

pH value	Ratio bola:DPPC (*n*/*n*)	First transition (*T*_1_)			Second transition (*T*_2_)			Third transition (*T*_3_)		
		*T*/°C	Fwhm/K	Δ*H*/kJ mol^−1^	*T*/°C	Fwhm/K	Δ*H*/kJ mol^−1^	T/°C	Fwhm/K	Δ*H*/kJ mol^−1^
5.0	1:4	31.6	1.8	1.0	41.8	1.0	20.5	85 ^1^	n.a.	n.a.
	1:1	32.7	1.6	8.0	41.5	1.7	11.3	89.5	5.3	21.5
7.7	1:4	32.2	1.8	1.5	41.8	1.0	19.3	^2^	n.a.	n.a.
	1:1	32.8	1.5	6.3	41.5	1.8	9.4	90.5	4.8	13.0
10.0	1:4	32.5	1.7	1.6	41.6	1.2	22.3	^2^	n.a.	n.a.
	1:1	32.2	1.6	6.2	40.8	2.1	10.8	86.2	6.7	23.0

^1^ very broad peak; ^2^ decrease in *C*_p_ value, no peak formation; n.a. = not assignable.

## Supplementary Materials

The following are available online at www.mdpi.com/2073-4360/9/11/573/s1, Figure S1. MS data (negative mode) of **Me_2_PE-Gly(2C16)C32-OBn**. Figure S2. MS data (positive mode) of **Me_2_PE-Gly(2C16)C32-OBn**. Figure S3. ^1^H-NMR data of **Me_2_PE-Gly(2C16)C32-OBn**. Figure S4. ^13^C-NMR data **Me_2_PE-Gly(2C16)C32-OBn**. Figure S5. MS data (positive mode) of **Me_2_PE-Gly(2C16)C32-OH**. Figure S6. ^1^H-NMR data of **Me_2_PE-Gly(2C16)C32-OH**. Figure S7. ^13^C-NMR data of **Me_2_PE-Gly(2C16)C32-OH**.
