# Supplementary material for: An Asymmetrical Glycerol Diether Bolalipid with Protonable Phosphodimethylethanolamine Headgroup: The Impact of pH on Aggregation Behavior and Miscibility with DPPC
Source: Polymers (Basel). 2017 Nov 3;9(11):573. doi: 10.3390/polym9110573 (PMC6418739; doi:10.3390/polym9110573)
Supplement: Supplementary file 1 [file polymers-09-00573-s001.pdf]

polymers

Supplementary Materials

An Asymmetrical Glycerol Diether Bolalipid with Protonable Phosphodimethylethanolamine Headgroup: The Impact of pH on Aggregation Behavior and Miscibility with DPPC

Thomas Markowski [1,†], Sindy Müller [2,†], Bodo Dobner [1], Annette Meister [3,4], Alfred Blume [3] and Simon Drescher [2,*]

[1] Institute of Pharmacy—Biochemical Pharmacy, Martin Luther University (MLU) Halle-Wittenberg, Wolfgang-Langenbeck-Strasse 4, 06120 Halle (Saale), Germany; thomas.markowski@pharmazie.uni-halle.de (T.M.); bodo.dobner@pharmazie.uni-halle.de (B.D.)
[2] Institute of Pharmacy—Biophysical Pharmacy, MLU Halle-Wittenberg, Wolfgang-Langenbeck-Strasse 4, 06120 Halle (Saale), Germany; sindy.lindner@pharmazie.uni-halle.de
[3] Institute of Chemistry—Biophysical Chemistry, MLU Halle-Wittenberg, von-Danckelmann-Platz 4, 06120 Halle (Saale), Germany; annette.meister@chemie.uni-halle.de (A.M.); alfred.blume@chemie.uni-halle.de (A.B.)
[4] Institute of Biochemistry and Biotechnology, MLU Halle-Wittenberg, Kurt-Mothes-Strasse 3, 06120 Halle (Saale), Germany
* Correspondence: simon.drescher@pharmazie.uni-halle.de; Tel.: +49-345-55-25196
† Thomas Markowski and Sindy Müller contributed equally to this work.

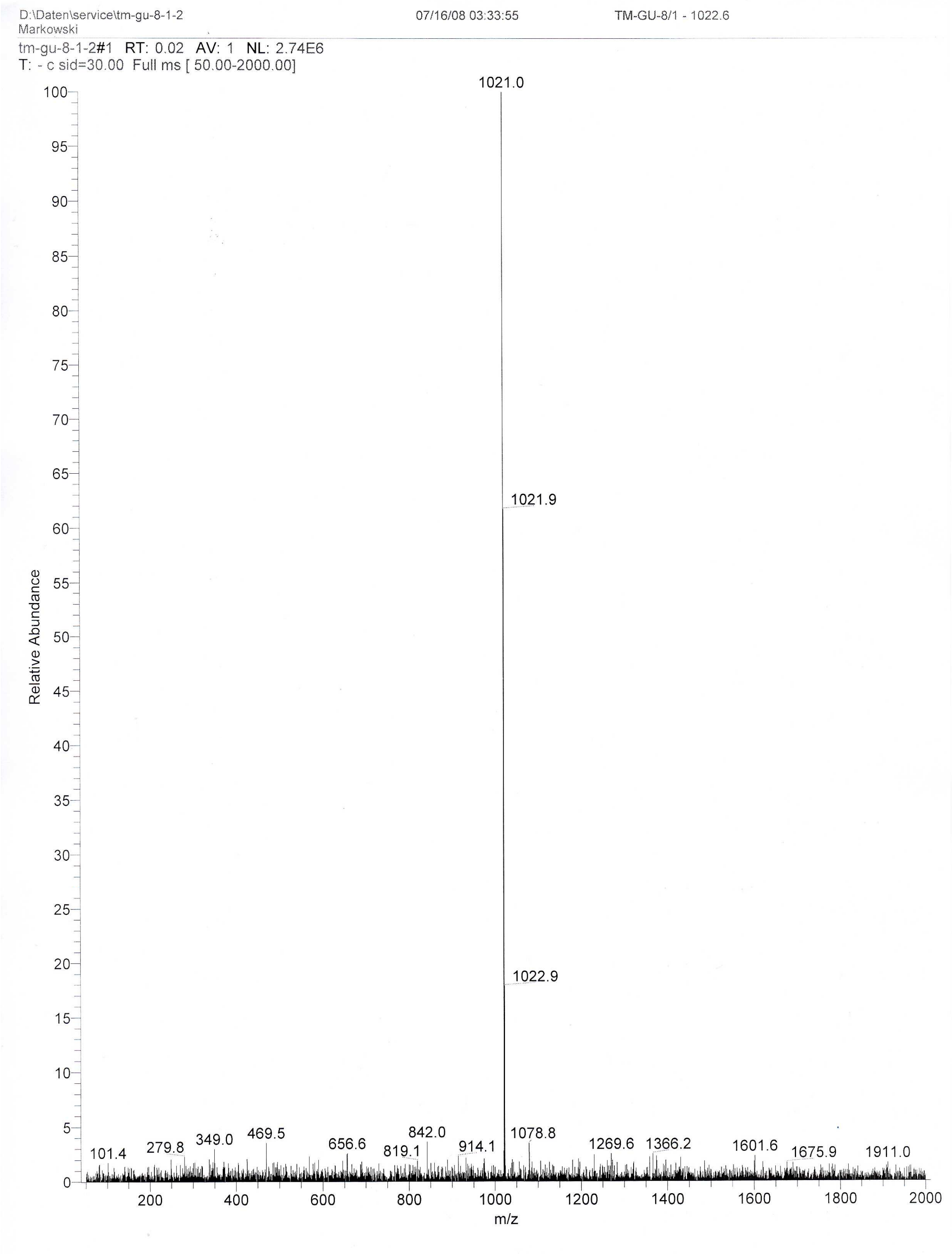

Figure S1. MS data (negative mode) of **Me_2PE-Gly(2C16)C32-OBn**.

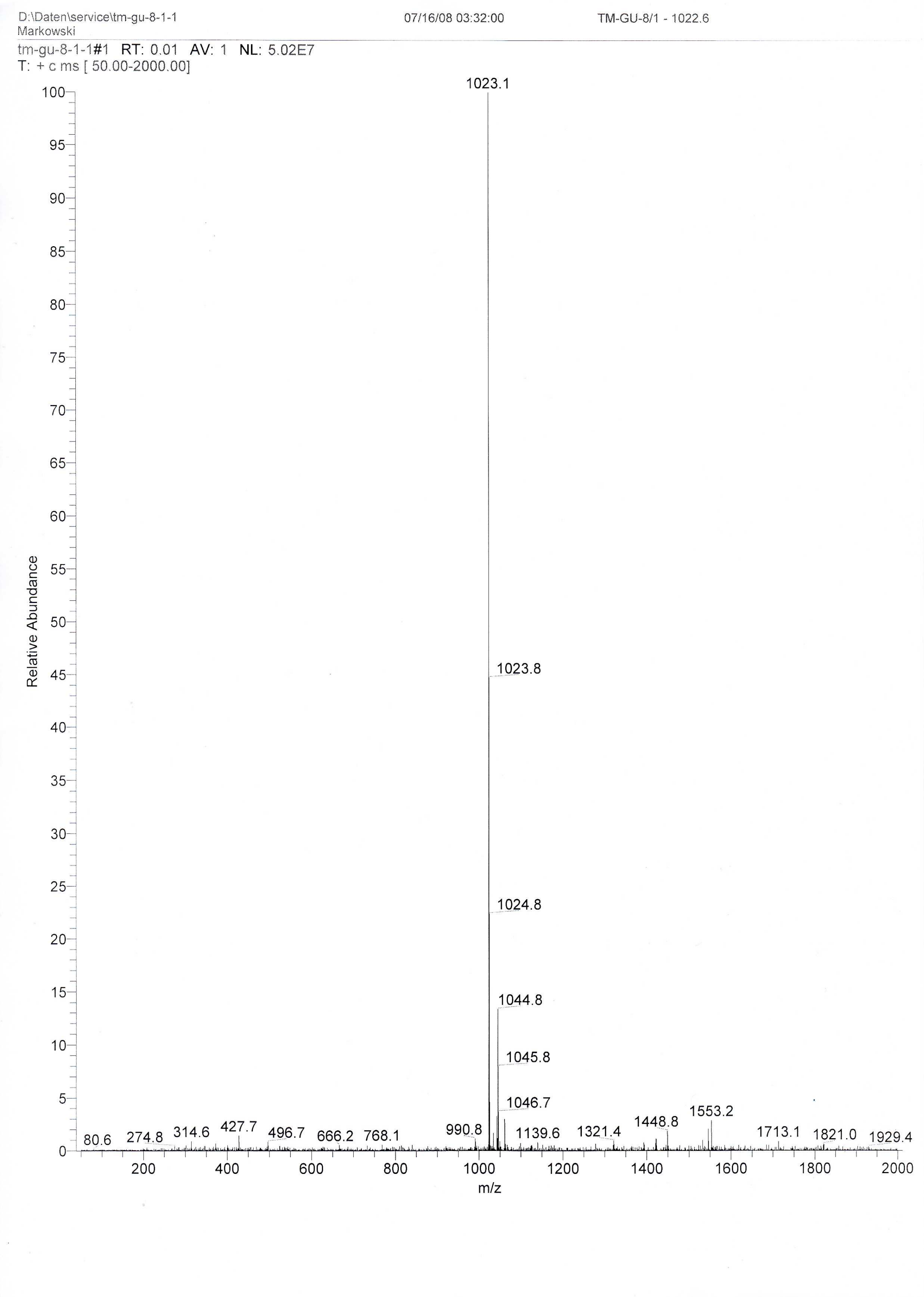

Figure S2. MS data (positive mode) of **Me2PE-Gly(2C16)C32-OBn**.

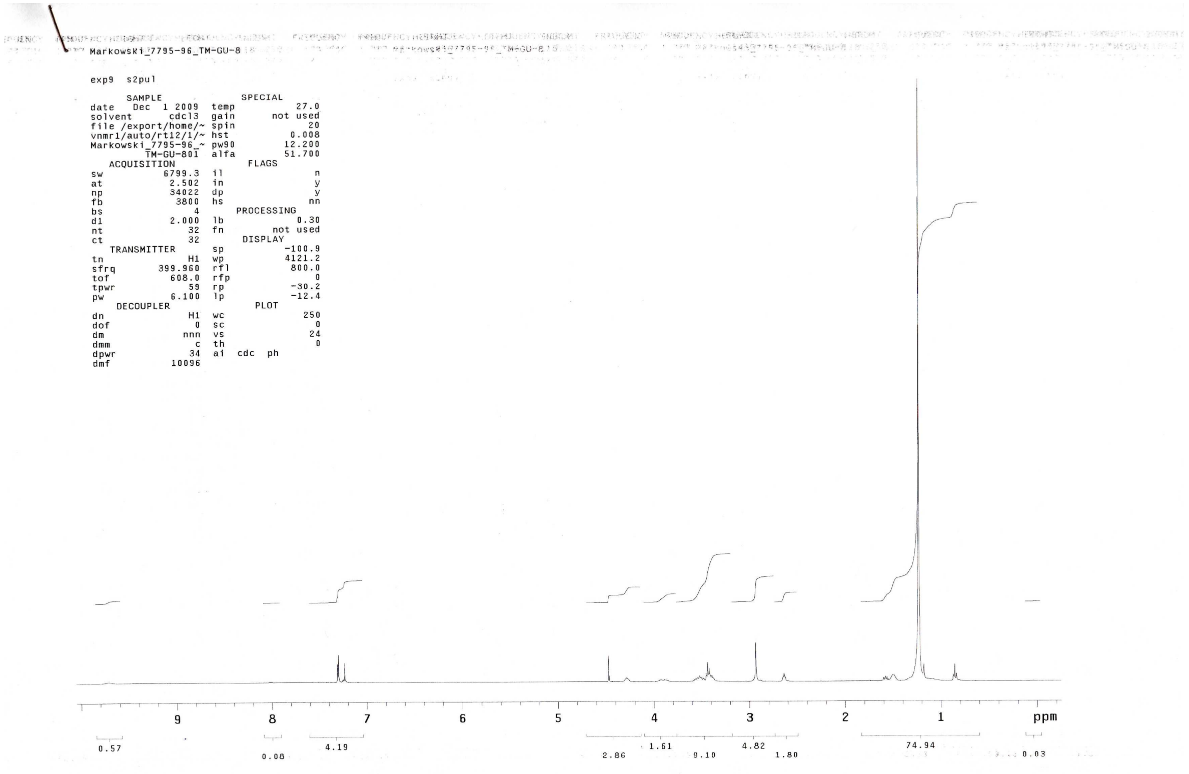

Figure S3. ^{1}H-NMR data of **Me2PE-Gly(2C16)C32-OBn**.

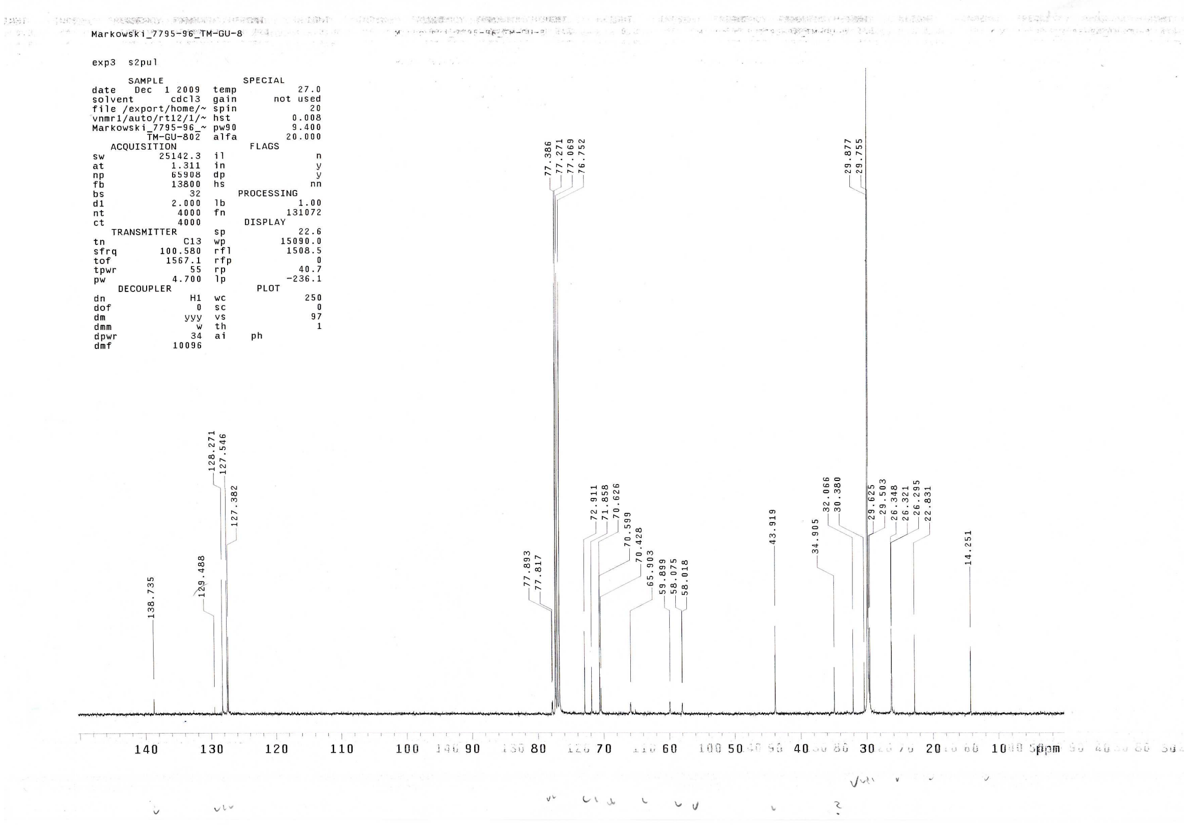

Figure S4. ^{13}C-NMR data **Me2PE-Gly(2C16)C32-OBn**.

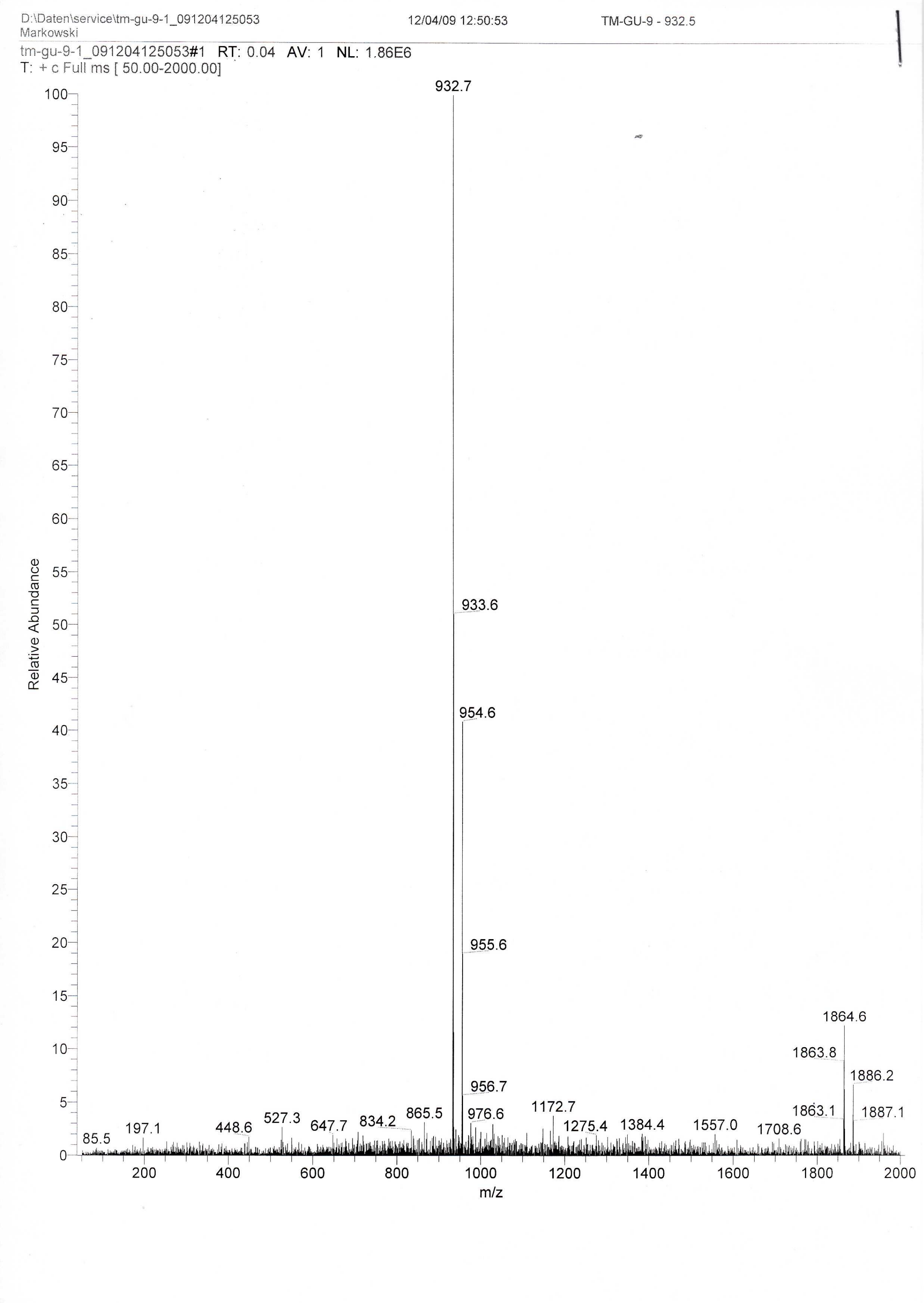

Figure S5. MS data (positive mode) of **Me2PE-Gly(2C16)C32-OH**.

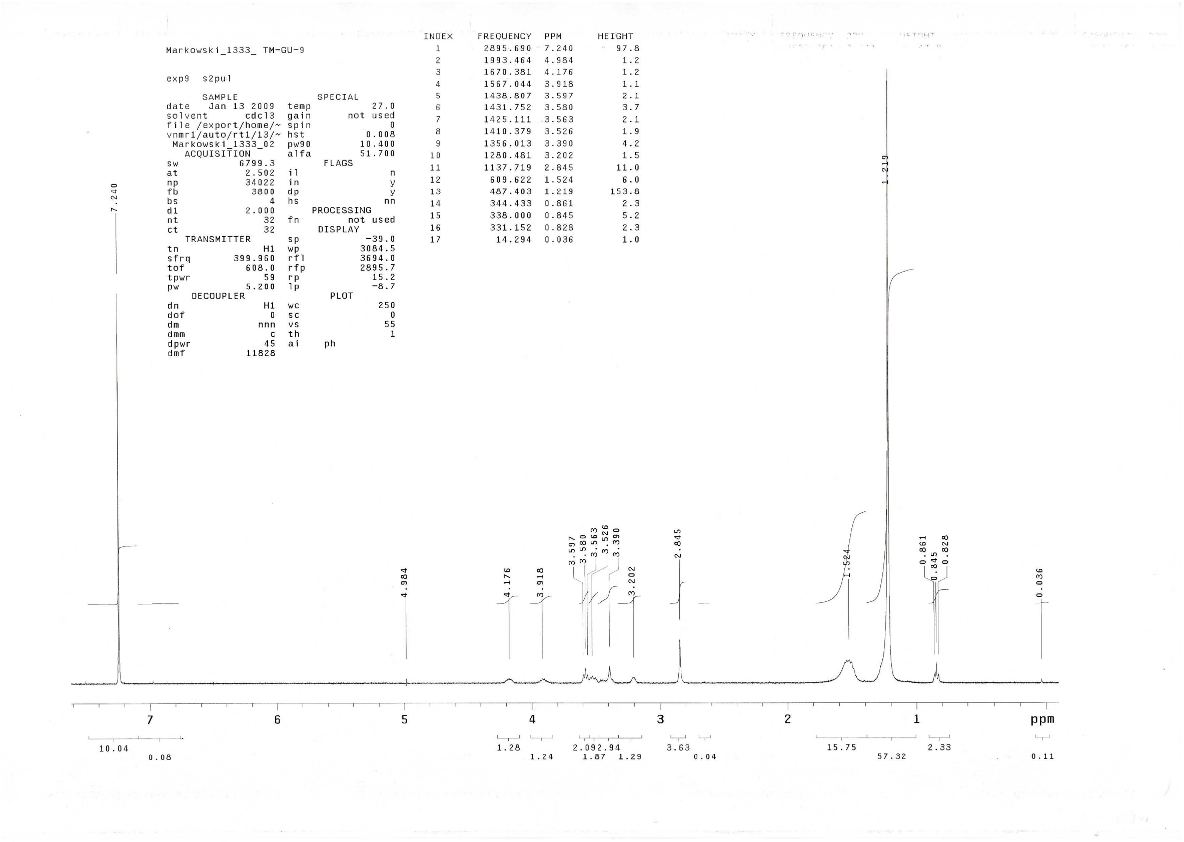

Figure S6. ^{1}H-NMR data of **Me2PE-Gly(2C16)C32-OH**.

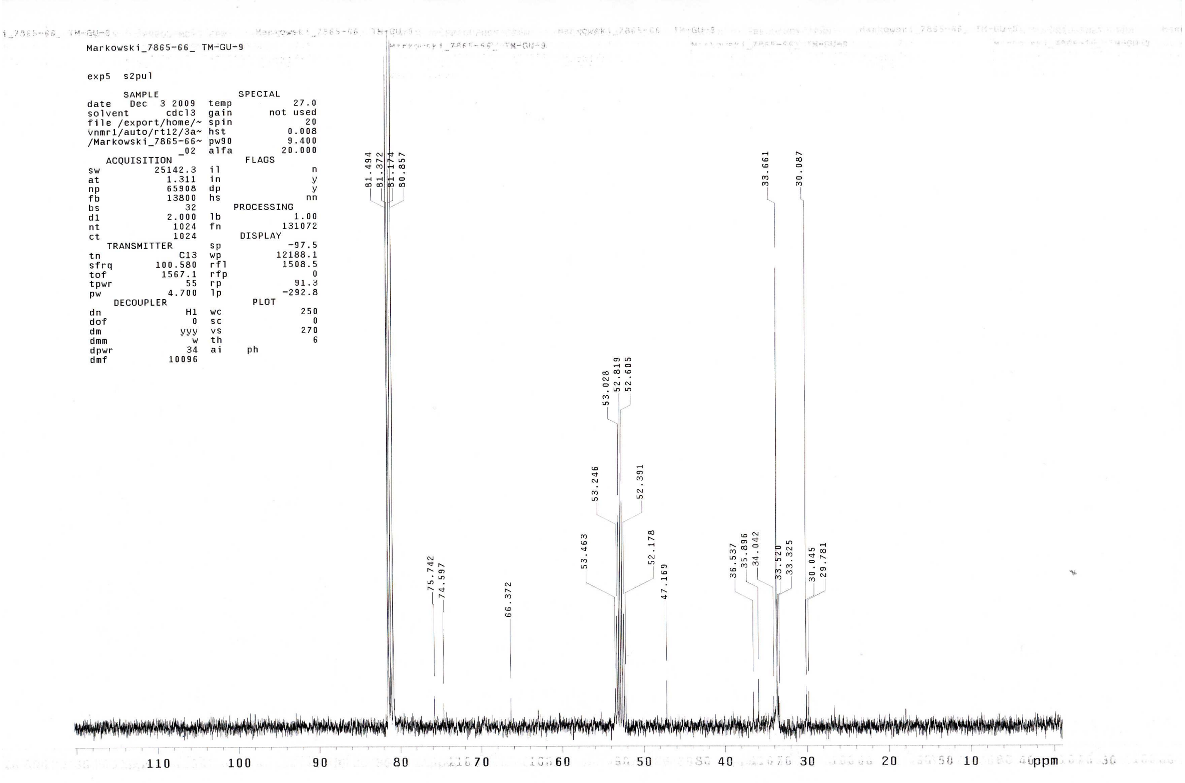

Figure S7. ^{13}C-NMR data of **Me2PE-Gly(2C16)C32-OH**.
